# Bilateral Abductor Paralysis of the Larynx

**Published:** 1903-11

**Authors:** Adolph H. Urban

**Affiliations:** Buffalo, N. Y.; Clinical instructor of diseases of the nose, throat and ear at the University of Buffalo, Medical Department and laryngologist and otologist to the University and German Hospital Dispensaries


### Bilateral Abductor Paralysis of the Larynx.

By ADOLPH H. URBAN, B. S., M. D., Buffalo, N. Y.,

Clinical instructor of diseases of the nose, throat and ear at the University of Buffalo, Medical Department and laryngologist and otologist to the University and German Hospital Dispensaries.

BILATERAL paralysis of the abductors of the larynx is a relatively rare condition and as few cases have been observed, much less reported, I consider the following one, which I shall dwell on more fully later, of sufficient importance to be published.

Loss of function of the dilators of the larynx is clinically observed as paralysis of the vocal cords and as such is seen by means of the laryngoscope. The muscles affected in this condition are the posterior cricoarytenoid, which separate the vocal cords. In briefly alluding to the nervous aspects of this variety of laryngeal stenosis, we find that opening of the glottis during inspiration, accomplished by the activity of these muscles, is presided over by a distinct medullary center. The motor nerve-supply is furnished by the abductor filaments of the inferior laryngeal nerve, the latter being the recurrent trunk of the pneumogastric.

Although a brief summary of this subject may be more academical than clinical it is, nevertheless, interesting. It was only in the year 1880 that we find such a careful observer as Störk[1] affirm that this was one of the rarest of laryngeal paralyses. Previous to this time in 1876, Riegel[2] in his work on Respiratory Paralysis had mentioned this condition as bilateral paralysis of the abductors of the vocal cords. A few cases had also been reported during this period by Von Ziemssen[3], Bosworth[4] and others. The first careful description of this condition was in 1878 by Semon[5]. He evolved his doctrine of the greater vulnerability of the abductor filaments of the recurrent nerve to injury from disease or trauma. This was primarily and entirely propounded and based on careful clinical observation and not on experimental researches as heretofore. In 1881, Semon[6] elaborated on this theory. At this period Rosenbach[7], working independently, made his own researches and arrived at the same general conclusions. They established the fact, now recognised as Semon's well-known law[8], "that paresis confined to the abductors is commonly, if not always, due to organic changes in the pneumogastric or recurrent trunk, or in the medulla." In so many words, they declared that the abductor muscles were regularly the first and often the only muscles affected in progressive organic laryngeal paralyses, and that the paralysis of the adductor is always secondary to paralysis of the abductor muscle.

Meanwhile Jeleneffy[9], Krause[10], and several other subsequent writers had advanced the "contracture theory" which claimed that the spasm either with or without paralysis of the abductors was due to various stimuli, irritating either the nerve trunks or their cerebral centers. In other words, they claimed that instead of paralysis of the abductors, it was clonic spasm of the abductors that produced the stenosis. From this it will be observed that this latter view tended to invalidate the law formulated by Semon, Rosenbach and others.

The explanation, however, of the Semon-Rosenbach theory was more or less hypothetical until Risien Russell[11], [12], as well as Onodi, established the fact that the nerve fibers for adduction and abduction of the vocal cords run in distinct bundles during the whole course of the nerve trunk, and that the fibers for the abductors are for the most part at the inner tracheal, while those for the abductors are at the outer side of the recurrent. More recently Grabower[13], by a special method of staining, has found a marked difference between the nerve endings of the abductor and adductor filaments in these muscles and believes that further future investigations will explain this abductor proclivity. Horseley[14] and others supported the Semon school in their contributions to this subject. At a still later date, 1898, Grossman[15] has endeavored to demonstrate by a series of experiments that both the Semon law and the Krause "spasm" theory could not be maintained, but his view, apparently, is not supported by adequate clinical observation.

After many years of controversy it might be stated definitely that the Semon view has held the balance of power and is at present the generally accepted one. It is therefore supposed that the median position of the vocal cords denotes interference by disease or trauma with a separate abductor nerve bundle, either in the recurrent nerve or its cerebral origin. Again, if the abductor muscles are more liable to disease or, in other words, offer less resistance to injury or central lesions, it has been correctly inferred that they are the last to recover when a paralysis of the recurrent is gradually improving. To affirm this, Elsberg[16] reports a case in which a bilateral recurrent paralysis terminated in a bilateral abductor paralysis, necessitating tracheotomy to avoid suffocation. This greater recuperative power of the adductors has also been exemplified in cases cited by Semon[17], Makins[18], Williams[19], Gleitsmann[20].

It may be well to remember that the posterior cricoarytenoid muscles are in a relatively exposed location in the larynx and in ceaseless activity. In quiet breathing this latter phase is not readily apparent to the eye. Although we have now noted that the abductor filaments run separately through the nerve trunk, nevertheless the fact remains that they are not in the most exposed position, nor would the anatomical location of these dilator muscles afford a satisfactory explanation of the actual condition of affairs—namely, that the abductors yield more readily to paralysing influences. The true pathogenesis of this special proclivity of the dilators of the larynx is, therefore, as yet a more or less obscure problem. As in other paralyses the lesions causing bilateral abductor paralysis may be either central or peripheral and are always organic.

The most frequent central cause is tabes dorsalis. According to Lennox Browne[21] this condition "is essentially the product of tabes." Others in their order of frequency are progressive bulbar paralysis, cerebral hemorrhages and softenings, cerebral lues, malignant tumors, abscesses and aneurisms producing compression of bulb, amyotropic lateral sclerosis, syringomyelia. Lennox Browne[22] states that "disseminated sclerosis and progressive muscular atrophy may be thought of, but since these tend to complete recurrent paralysis, it is a rare coincidence to detect their initial effects (*i. e.,* abductor paralysis) simultaneous on both cords." Considerable doubt still exists as to whether diphtheria can be an etiological factor centrally by producing nerve disturbances through the medulla. Luc[23] admits this, although he believes that it attacks the peripheral nerves much more frequently.

Amongst the peripheral causes may be mentioned tumors of the neck, especially thyroid growths. Tumors at the base of the brain are in order of frequency, gummata, carcinoma and sarcoma. Other lesions are pachymeningitis, tumors of the mediastinum, aneurisms, especially of the aorta, carcinoma of the esophagus (located high up), trauma, enlarged peribronchial lymphatic glands, pericardial effusions, pleuritic adhesions; these must of necessity involve the recurrent nerve bilaterally during its course to produce,—abductor paralysis duplex. Rheumatism and alcoholism as etiological factors are still problematical. The most frequent peripheral causes, however, are infectious diseases as well as toxic poisonings. Amongst the former might be mentioned influenza, diphtheria, pneumonia, the acute exanthemata, syphilis and typhoid. Toxic influences are chiefly lead; there are also instances of paralysis arising from copper, antimony, phosphorus, arsenic and atropin. Occasionally, we must deal with simple anginas, catarrhal conditions and exposure to cold. Impaction of foreign bodies, notwithstanding their removal, has caused this stenosis. Bruggisser[24] reports the case of a man, aged 24, with a rubber plate containing two false teeth in the larynx. The obstruction was removed on the eighth day but was followed by a complete bilateral abductor paralysis; tracheotomy had to be performed to relieve the condition. Hysteria, though purely functional, is reported to have brought about the same result; conclusive evidence, however, was lacking.

During inspiration the laryngeal mirror in bilateral abductor paralysis shows the vocal cords lying motionless near the median line instead of being separated by the action of the dilators. In expiration the cords are forced asunder by the current of outgoing air. The phonatory image is not affected and hence in ordinary conversation the voice is influenced little, if any. In this stenosis the dyspnea increases during the hours of sleep, owing to the fact that the dilator muscles of the larynx are during that period no longer under the control of the will. The diagnosis of the condition is generally a not very difficult one. In fact, as Riegel[25] has stated, in a complete paralysis the diagnosis can often be made without the aid of a mirror. However, there have been well marked tabetic cases in which the inspiratory dyspnea was totally absent; in such instances the eye was a diagnostic necessity.

In objective appearances severe spasm of the glottis is the most frequent source of error in diagnosis. In contradistinction to paralysis, however, the dyspnea in stridor usually improves at night; the cords are not held rigidly in the median line but waver more or less, causing a constant variation in the amount of adduction; in addition, laryngeal spasm generally attacks very young, neurotic children; again, if examined closely, the cords at the end of expiration will be seen to separate of their own accord, which does not happen in paralysis. Ankylosis of the cricoarytenoid joints, due especially to carcinoma of the esophagus, tubercular or leutic laryngitis, may at times be difficult to differentiate from the lesion under discussion. Perichondritis of the cricoid, injury to the cricoarytenoid articulation, and rarely congenital ankylosis may be thought of. It is also to be remembered that a cicatricial contraction on the posterior aspect of the larynx between the arytenoid cartilages, may produce an image simulating this condition (Sidlo)[26]. Again, a malignant new-growth in the subglottic space (Semon)[27], or an abscess located between the posterior plate of the cricoid and the postici muscles (Kuttner)[28] may cause a rigid median position of the vocal cords. Without entering into the subject, I might mention that the determination of the focal lesion causing the bilateral paralysis is of necessity the most important and very often the most difficult question.

This form of laryngeal palsy follows a very variable course. In some cases a certain degree of double sided paresis may remain stationary for years, in others we can observe the gradual transition to a complete paralysis. Again, an absolute loss of function may not alter its aspect for a long period, while another may pass into a more or less complete recurrent paralysis. In many instances, particularly in tabes, the condition is absolutely symmetrical, while in others, both due to central (*e. g.*, pachymeningitis) or peripheral (*e. g.*, carcinoma of the esophagus) causes, we find a complete, unilateral posticus paralysis, in fact, occasionally a total recurrent affection, whilst the other cord exhibits only a beginning loss of power. Thus we see that not only does the laryngoscopic image, but the voice and respiratory symptoms vary within a wide range.

The prognosis naturally depends entirely upon the causal effect. Occasionally, however, we see extreme cases of tabic and bulbar origin go on for years without leading to consequences of more serious import. On the other hand, in some peripheral varieties of chronic, progressive character we must give an unfavorable prognosis for eventual recovery. In diphtheritic laryngeal palsies, such as the following case is an exhibition of, the prognosis is generally comparatively favorable.

Although it is not within my power to add anything new in regard to the etiology or treatment, yet the case to be reported only again confirms the law that of the motor fibres of the larynx the abductors are the first to succumb to any lesion. It also exhibits very forcibly the slow recuperative powers of the laryngeal dilators.

The case in question which came under my care at the German Hospital in New York city, was a boy aged 15. He was admitted on the afternoon of the 26th of February, 1900. I saw the patient immediately after his arrival. The boy, who was suffering from dyspnea, had recovered from a serious attack of diphtheria six weeks previously; otherwise his condition had always been good. The family history was negative. For the last three weeks the respiration had been very noisy. This was especially so during inspiration, which was a distinctly fatiguing effort. When the patient was sleeping or making any exertion this noisy breathing became worse. Deglutition had not been interfered with nor was there any tendency towards the regurgitation of fluids through the nose. No history of any sensory symptoms was given. Upon examination it was found that the boy, who was very anxious and restless, was of good physique, but somewhat pale and emaciated. The physical examination was absolutely negative, the respirations were 27, and the pulse was 96 and regular. Though the chest was well-developed there was increased depression in the suprasternal and supraclavicular fossæ. There was no abdominal pain or vomiting, the bowels were constipated, the urine exhibited only a slight albuminuria. No eye symptoms were present; the hearing was unimpaired; the nares and pharynx were free. Inspection of the neck revealed nothing abnormal. Neither the sphincters nor the muscles of neck, face, palate, trunk and extremities were involved. Direct protrusion of tongue was possible ; gait and reflexes were not influenced. The examination of the larynx showed the vocal cords lying almost in apposition in the median line. In expiration they seemed to be pushed apart by the current of the outgoing air, while in inspiration they approached each other closely, causing the chink of the glottis to become a mere cleft. There was no perceptible hyperemia or infiltration of any part. There was no anesthesia of larynx or pharynx. The voice was only slightly affected, being somewhat coarse and weakened, the speech being free as long as the inspired air in the lungs lasted after which the patient paused and made a forced inspiration accompanied by a very marked elevation of the shoulders. There was absolutely no pain; the expression of the face was extremely anxious.

In the presence of this characteristic laryngeal condition and the subsequent course of the disease, there was hardly any doubt but that we were dealing with a post-diphtheritic bilateral abductor paralysis of the vocal cords. Operation was decided upon and tracheotomy fixed for two days later. During the following night the inspiratory dyspnea and noisy, stridulous breathing became more pronounced. The next morning the condition became much worse. Preceded by terrible anxiety and apprehension but without convulsions, the patient suddenly became very cyanotic, ceased breathing and lost consciousness; the pulse became very rapid, at times scarcely perceptible; death from suffocation seemed imminent. Without any anesthesia and while artificial respiration was being resorted to, I immediately performed tracheotomy, the incision being made below the isthmus of the thyroid. No difficulty was experienced.

After the insertion of the tube, which occupied but a few moments, and during continued artificial respiration the patient slowly began to breathe freely and easily. The cyanosis gradually disappeared and the pulse slowly improved. Rest was enjoined and rectal feeding resorted to. The boy was kept warm and made an uncomplicated recovery from the operation. Two days later the patient was nourished per mouth on a fatty, nutritious diet and placed on strychnine, iron and arsenic. Electricity, in the form of the galvanic current was employed. The patient rapidly gained in weight and appearance and was discharged from the hospital on March 18, 1900. The tube has been worn constantly ever since, *i. e.,* for a period of three and one-half years and has given entire relief. At the present writing the general condition is much improved. By placing the finger on the mouth of the tube the boy can breathe with but little difficulty for several hours when, however, the respiration becomes slightly tedious and labored. The speech is clearer and stronger than formerly. The boy feels fairly comfortable, having become accustomed to the constant wearing of the tube (to be dispensed with at an early date.)

In discussing the operative treatment of this case Semon's indication[29] for prophylactic tracheotomy, namely, "when the greater widening of the glottic space can not be brought about by suitable treatment," would have been present whatever the course of the condition. Intubation would only have been a temporary expedient and it would have been necessary to follow it very soon by tracheotomy. Permanent intubation certainly seems entirely irrational. As regards thyrotomy and excision of a vocal cord[30], this surely cannot be compared to tracheotomy, especially as the effect of the former operation not only produces permanent complete aphonia, but practically defeats its intended object by the consequential cicatricial formation in the larynx.[31] Section of the recurrent nerve, advised by Geronzi[32], but opposed by Ruault[33], to attain the so-called cadaveric position could only possibly be justified in cases in which the etiological factor is steadily progressive and would certainly never be the indication where there is any prospect at all of recovery.

As regards the medicinal treatment the most useful and valuable drugs are strychnine, arsenic, iron and phosphorus. Electricity is a resource of undoubted value and at that time in our case the galvanic current employed by both the extra- and endolaryngeal method was the most beneficial for the reason that the palsy was a comparatively recent one. Since then both the galvanic and faradic current have been alternately employed.

The history of the case pointed undoubtedly to diphtheria, and as no conjoined disorders of any nerve centre were present, we were evidently dealing with a chronic localised degeneration of the abductor nerve-filaments in, as well as an atrophy of, the laryngeal dilator muscles. The direct cause of this was a peripheral toxic neuritis. The main point of clinical interest,—the scientific side of the question having been sufficiently discussed,—is the fact that we had a purely primary bilateral abductor paraysis of the vocal cords of the larynx. Again, the same had continued practically stationary for over three years and only recently (during the past year) has the condition shown a tendency to eventual recovery. As diphtheritic paralytic sequelæ generally recover in from three to six months, this case certainly presented an exception to the general rule and was most probably due to an excessive focal toxic condition. This course of the lesion also precluded the possibility of mechanical alterations in or about the cricoarytenoid joint. Another interesting point is the sudden aggravation of the symptoms necessitating immediate tracheotomy. Cases with similar crisis as to sudden danger to life have been reported amongst others by C. W. Hartwig[34] and Semon[35].

BIBLIOGRAPHY.

1. Störk: Klinik der Krankheiten des Kehlkopfes. 1880. P. 97.
2. F. Riegel: Volkmann's Samml. Klin. Vorträge, No. 95. 1875.
3. v. Ziemssen: Deutsche Archiv. f. Klin. Medizin. Bd. XVIII. 1876.
4. Bosworth: Diseases of Throat and Nose.
5. F. Semon: Transactions of the Clin. Soc. of London, Vol. XI., P. 141.
6. F. Semon: Archives of Laryngology, July, 1881.
7. O. Rosenbach: Breslauer Aerztl. Zeitschrift, 2, 3. 1880.
8. McBride: Diseases of the Throat. 1900.
9. Jeleneffy: Berliner Klinische Wochenschrift, No. 26, 34 *seq.* 1888.
10. Krause: Virchow's Archiv., No. 98. 1884. P. 294.
11. Risien Russell: British Medical Journal, June 18, 1892.
12. Risien Russell: Proceedings Royal Society, Vol. LI. 1892.
13. Grabower: Archiv. für Laryngologie, Vol. VII., P. 128.
14. Horsely: British Medical Journal, Dec. 21, 1889.

15. MacIntyre: Journal of Laryngology, May, 1898.
16. Elsberg: Philadelphia Medical Times, July 30, 1881.
17. F. Semon: Proceedings of the Royal Institute, Vol. XIII. 1891.
18. G. Makins: British Medical Journal, May 16, 1896.
19. P. Watson Williams: Diseases of the Upper Respiratory Tract. 1901. P. 238.
20. J. W. Gleitsmann: The Laryngoscope, October, 1901. P. 290.
21. Lennox Browne: The Throat and Nose and their Diseases. 1899.
22. Lennox Browne: The Throat and Nose and their Diseases. 1899.
23. Schmidt: Krankheiten der Oberen Luftwege. 1897. P. 683.
24. Bruggisser: Corresp. Blatt. f. Schweizer Aerzte, Heft 15. 1900.
25. Riegel: Volkmann's Samml. Klin. Vorträge, No. 95. 1875.
26. Sidlo: Wiener Medicinische Wochenschrift, No. 26, 27, 29. 1875.
27. P. Heymann: Handbuch der Laryngologie und Rhinologie, 1 Band. 1898.
28. Schmidt: Krankheiten der Oberen Luftwege. 1897. P. 708.
29. F. Semon: Some Thoughts on the Principles of Local Treatment in Diseases of the Upper Air Passages. 1902. P. 77.
30. Greville Macdonald: Schmidt, Krankheiten der Oberen Luftwege. 1897. P. 716.
31. P. Heymann: Handbuch der Laryngologie und Rhinologie, 1 Band. 1898. P. 739.
32. Newcomb: Diseases of Throat. 1901.
33. H. Luc: Les Névropathies Laryngées. Paris. 1892.
34. C. W. Hartwig: Presbyterian Eye, Ear and Throat Charity Hospital, January, 1896.
35 F. Semon: Transactions Clinical Society of London, Vol. XI. 1878.

523 FRANKLIN STREET.

